# Delineation of Genotype-by-Environment interactions for identification and validation of resistant genotypes in mungbean to root-knot nematode (*Meloidogyne incognita*) using GGE biplot

**DOI:** 10.1038/s41598-020-60820-x

**Published:** 2020-03-05

**Authors:** Bansa Singh, Arpita Das, A. K. Parihar, B. Bhagawati, Deepak Singh, K. N. Pathak, Kusum Dwivedi, Niranjan Das, Nishi Keshari, R. L. Midha, Raju Kumar, Aditya Pratap, Vaibhav Kumar, Sanjeev Gupta

**Affiliations:** 1 ICAR- Indian Institute of Pulses Research, Kanpur, 208024 Uttar Pradesh India; 2 Bidhan Chandra Krishi Viswavidyalaya, Mohanpur, Nadia, West Bengal India; 3 Assam Agricultural University, Jorhat, Assam India; 4 ICAR- Indian Agricultural Statistics Research Institute, New Delhi, 110 012 India; 5 Rajendra Prasad Central Agricultural University, Pusa, Samastipur, Bihar India; 6 Chandra Shekhar Azad University of Agriculture & Technology, Kanpur, 208 002 Uttar Pradesh India; 7 Orissa University of Agriculture & Technology, Bhubaneswar, Orissa India; 8 Rajasthan Agricultural Research Institute, Durgapura, Jaipur, Rajasthan India; 9 All India Coordinated Research Project (AICRP) on MULLaRP, ICAR- Indian Institute of Pulses Research, Kanpur, 208 024 Uttar Pradesh India

**Keywords:** Genetics, Plant sciences

## Abstract

Susceptibility to root-knot nematodes (*Meloidogyne* spp.) is one of the major factors limiting mungbean production in South and South-East Asia. Host-pest-environment interaction in mungbean and root-knot nematode (*M*. *incognita*) was investigated in multi-location field evaluation using 38 promising mungbean genotypes extracted from initial evaluation of 250 genotypes under sick plots considering second stage freshly hatched juvenile as inoculants. The extent of environmental and genotype-by-environment interactions (GGE) was assessed to comprehend the dynamism of resistance and identification of durable resistant mungbean genotypes. Among environmental factors, nematode activity was highly influenced by rainfall and minimum temperature. The GGE biplot and multiple comparison tests detected a higher proportion of genotype × environment (GE) interaction followed by genotype and environment on number of nematode galls, gall index and reproduction factor. The first two principal components (PCs) explained 64.33% and 66.99% of the total variation of the environment-centered gall scoring and reproduction factor data, respectively. The high GE variation indicated the presence of non-cross over interactions which justify the necessities of multi-location testing. Detection of non-redundant testing locations would expedite optimum resource utilization in future. The GGE biplot analysis identified genotypes such as PM-10-12, IPM-410-3 and NVL-641 as the outperforming and desirable genotypes with durable resistance against *M*. *incognita* which can be exploited in mungbean breeding programmes globally. On the contrary, the highest gall scoring and reproduction factor were recorded in genotype IPM-9901-8. Computation of confidence interval (CI) at 95% level through bootstrapping increased precision of GGE biplot towards genotype recommendation. Furthermore, total phenol content, ascorbic acid, phenlylalanine ammonia lyase (PAL) and polyphenol oxidase (PPO) activities were also higher in identified resistant genotypes and this information would be useful for devising mungbean breeding strategies in future for resistance against root-knot nematodes.

## Introduction

Mungbean (*Vigna radiata* L.Wilczek) is an important nutritious pulse crop playing a crucial role in combating malnutrition among vegetarian population of South and South-East Asia (SEA), Africa, South America and Australia^1^. Notwithstanding, the increase in area and production, productivity of this crop is quite low as compared to other pulse crops^2^. There are several biotic and abiotic stresses including root-knot nematodes which are responsible for low productivity of mungbean^3,4^. Root-knot nematodes (*Meloidogyne* spp.) mainly affect mungbean production in South, East, and Southeast Asia, Africa and South America^5^ and cause 18-90 percent yield damage in congenial conditions^4,6–8^. These are serious parasites which attack wide varieties of crop plants including pulses and are responsible for substantial economic losses^9^. *M. incognita* is widely distributed and pest of several economically important crops causing huge losses. Besides pulses, *M. incognita* is capable of severely damaging a wide range of crops such as vegetables^10^, ornamental crops^11^, fiber crop cotton^12^, corn^13^ and weeds^14^.

More than 80 species of *Meloidogyne* have been reported in different parts of the world. Out of them, two species *M. incognita* and *M*. *javanica* have been reported in pulse- based cropping systems^15–17^. During the infective stage, root- knot nematodes feed on the epidermal cells of root and penetrate through the newly formed tissues present above the meristematic zone^18^. At the initial stage of the infestation by root-knot nematode in the plant tissue there is cell enlargement with rapid cell division in the pith and vascular bundle followed by transformation of cortex into gall^19^. Consequently, with the increment of infestation, growth and development of infested plants become stunted along with yellowing of leaf^20^. Extent of damage caused by root-knot nematode invasion varies with the initial nematode density present in the soil, host, cultural conditions and weather parameters like temperature, moisture etc. *Meloidogyne incognita* may complete its biological cycle in about 30 days when soil temperatures range from 25–30 °C^21^. Location and region having differences in soil conditions and environmental variations may influence the infectivity and losses caused by root knot nematodes in pulse crops, mainly mungbean.

Management of root-knot nematode is a vital endeavour for sustainable mungbean production. Besides, the application of available nematicides is also not a viable approach owing to the environmental hazard. Host-plant resistance for nematode especially *Meloidogyne* spp. has been reported in many crop species^22^. As huge amount of variability exists within the hosts and the pests, therefore, understanding of interaction for a particular system can be difficult and challenging^23^. Moreover, variability among the genotypes against particular pests arises due to differential genotypic response regarding enzymatic activity, since several stress related enzymes have been reported to be involved in defence reaction against pest^24^. The enzymatic action such as catalase and peroxidise led to scavenge the accumulation of H_2_O_2_ in tissue^25^. Catalase plays an important role in catabolism of H_2_O_2_. PAL is the entry point enzyme into phenyl propanoid metabolism, involved in production of phenolics and phytoalexins that prevent pest establishment^26^. Differential enzymatic activities in plants thus help in characterizing resistant and susceptible genotypes against parasitic nematodes.

Identification of stable and durable resistant sources of mungbean against root-knot nematode and their subsequent judicious utilization in resistance breeding programme would be an effective and efficient approach for sustainable production. Therefore, identification of stable genotypes having the minimum effect of environment in terms of yield and disease resistance performance assumes greater importance. Studies confirmed that GGE biplot analysis could be a wonderful tool for identification of the best resistant cultivar and the most virulent types that can be used to differentiate resistance level among cultivars^23^. Recently, GGE biplot has been deployed to assess genotypes with wide or specific adaptation related with resistance to different pathogens in many legumes^1,27–33^.

Indeed, GGE biplot technique is frequently used to judge the response of the genotypes but it is yet to be used for understanding of genotype response against nematode incidence across diverse locations. Hence, the present study was framed firstly, to identify stable and superior mungbean genotypes against *M*. *incognita* infestation which could be recommended for cultivation in specific root-knot nematode prevalent environments/ ecologies. Secondly, to assess the influence of environment on host-nematode response along with the grouping of various test locations into distinct mega-environments for identification of the ideal test locations for future testing.

## Results

### Genotypic response towards *M*. *incognita* infestation

Mungbean genotypes exhibited variable response against *M. incognita* infestation in the test locations. Analysis of variance of root-knot nematode incidence witnessed that the effect of genotype, environment and genotype × environment interactions were significant for all the parameters (Table 1). Mean Performance of the genotypes regarding *M*. *incognita* galls, gall index, final population and reproduction factor  is represented in Table 2. Over the locations considering all the parameters, GM-04-02 (8), PM-10-12 (28), NVL-641 (26), IPM-2-3 (14), PM- 09-11 (27), GGG-10-14 (3) and IPM-410-3 (16) were identified as resistant to moderately resistant genotypes. On the contrary, the highest gall scoring and reproduction factor were recorded in genotype IPM-9901-8 (18).

**Table 1:** Analysis of variance of root knot nematode incidence in 38 genotypes of mungbean evaluated at six locations in India.

Trait	MS			F value			% contribution of TSS		
	Geno	Env	Geno * Env	Geno	Env	Geno * Env	Geno	Env	Geno * Env
Gall Index	4.131	0.272	0.636	64.642**	4.250**	9.955**	41.28	8.53	42.99
Gall	6,435.9	1,795.2	1,063.8	265.0**	73.9**	43.8**	39.06	13.92	42.20
Population	295,066	84,947	66,252	186.2**	53.6**	41.8**	34.56	10.69	48.90
Reproduction Factor	7.37	2.13	1.65	186.0**	53.7**	41.8**	34.56	10.69	48.90

*P < 0.05, **P < 0.01.

**Table 2:** Details of parameters for nematode screening of 38 genotypes of mungbean at six locations.

Sl No.	Genotype	Gall Index	Galls	Population	Reproduction Factor
1	AKM-12-10	3.9	39.1	347.8	1.7
2	AKM-4	3.3	26.6	225.2	1.1
3	GGG-10-14	3.1	22.6	158.0	0.8
4	DGG-3	4.0	41.8	412.8	2.1
5	DGG-5	3.9	38.9	348.6	1.7
6	DGG-6	3.9	38.6	348.5	1.7
7	AKM-8802	4.0	40.2	366.7	1.8
8	GM-04-02	2.8	17.6	123.2	0.6
9	GM-11-02	3.6	43.0	301.0	1.5
10	HUM-1	4.0	55.4	422.7	2.1
11	HUM-27	3.8	48.1	336.7	1.7
12	IGKM-05-26-3	4.0	49.5	346.4	1.7
13	IPM-2-14	4.2	64.5	451.8	2.3
14	IPM-2-3	3.0	24.9	243.7	1.2
15	IPM-2K15-4	4.2	68.5	479.8	2.4
16	IPM-410-3	3.1	21.0	147.5	0.7
17	IPM 9901-6	4.0	54.3	380.1	1.9
18	IPM-9901-8	4.5	77.1	539.9	2.7
19	KM-2342	4.3	64.6	452.4	2.3
20	MH-275	3.7	34.8	309.6	1.5
21	MH-810	3.5	33.6	299.4	1.5
22	MH-934	4.0	44.9	399.7	2.0
23	ML-2056	3.8	43.2	384.3	1.9
24	ML-2333	3.7	34.8	309.5	1.5
25	NVL-516	3.8	41.4	368.9	1.8
26	NVL-641	3.0	26.3	144.8	0.7
27	PM-09-11	3.1	20.7	257.4	1.1
28	PM-10-12	2.9	20.0	140.4	0.7
29	PUSA-0672	4.0	59.8	418.4	2.1
30	PUSA-1371	4.0	57.2	400.2	2.0
31	PUSA-1471	4.0	56.9	398.1	2.0
32	PUSA-1472	3.5	34.9	271.5	1.4
33	RMG-1030	3.4	34.0	237.9	1.2
34	RMG-1028	3.4	33.0	323.0	1.6
35	SGC-20	3.2	29.3	287.2	1.4
36	TARM-1	3.9	40.2	377.3	1.9
37	TMB-45	3.2	31.1	304.7	1.5
38	VGG-05-006	3.6	37.8	370.9	1.9
Mean		3.7	40.8	327.3	1.6
C.D.		2.497	0.091	20.172	0.101

### Role of environmental parameters

**Figure 1:**
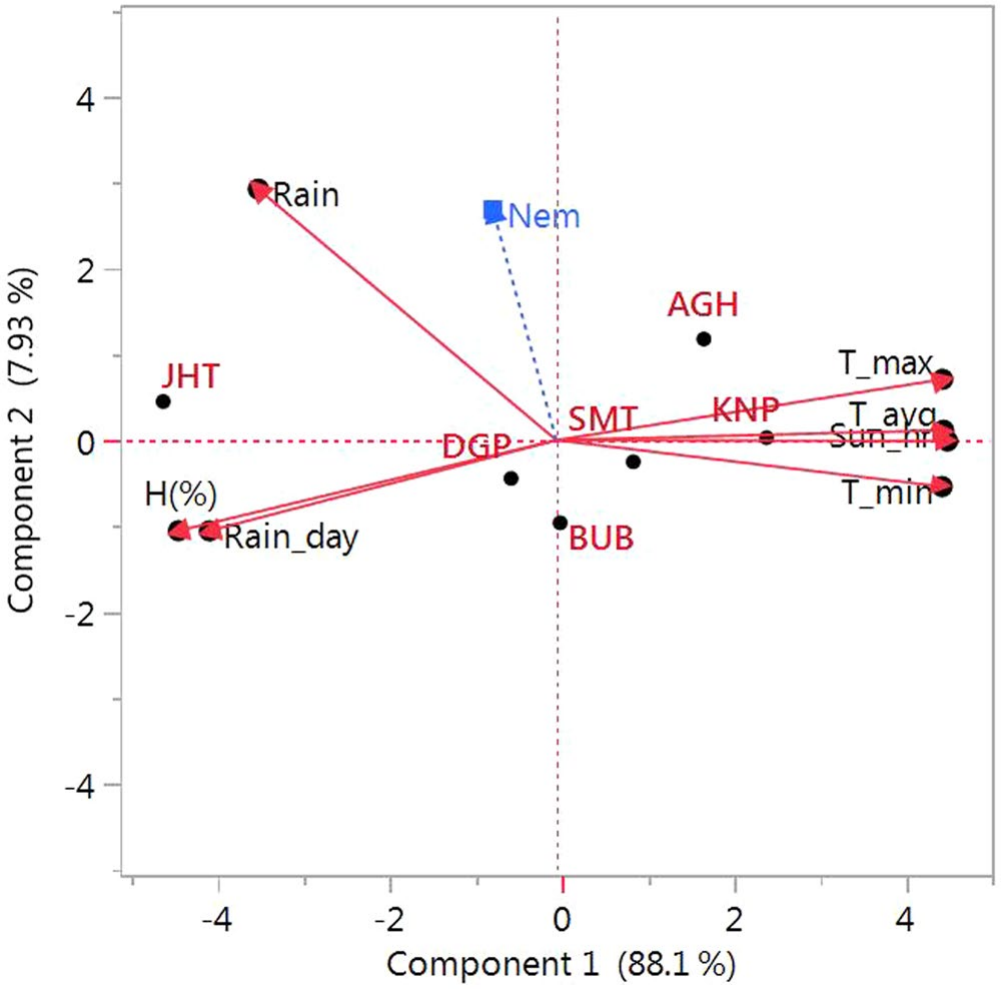
Principal Components on correlations biplot for *M. incognita* incidence in mungbean. Locations are: SMT, Samastipur; KN, Kanpur; BUB, Bhubaneswar; AH, Aligarh; DP Durgapura; JH, Jorhat.

Role of environmental parameters in root-knot nematode infestation across locations was illustrated by correlation (Table 3). Genotype × environment interaction towards nematode scoring must be elucidated considering the disease × environmental factor interaction. Nematode activity was highly influenced by rainfall (r = 0.4739) and minimum temperature (r = −0.2759). The prevalence of low temperature during early crop growth stage could hinder the invasion of *M. incognita*. The path analysis to represent the direct and indirect effect of environmental variables on nematode scoring in addition with their linear correlation revealed the highest positive direct effect by minimum temperature (2.779) followed by average rainfall (2.501) (Supplementary Table 1). The biplot view of weather variables and nematode scoring demonstrated a similar trend (Fig. 1). Acute angle was detected between nematode population and rainfall, which is indicative of a positive correlation, while rest of the variables portrayed obtuse angle and negative association with gall scoring.

**Table 3:** Correlation among the weather parameters and root-knot nematode gall index of 38 mungbean genotypes evaluated at six locations in India.

Env. variables	R.H. (%)	Max Temp (°C)	Min. Temp (°C)	Rainfall (mm)	Rainy day	Sunshine (hr)	Nematode gall index
Avg. Temp (°C)	−0.9651**	0.9832**	0.9808**	−0.7449*	−0.8355**	0.9944**	−0.1793
R.H. (%)		−0.9908**	−0.9092**	0.5775	0.8591**	−0.9547**	0.0864
Max Temp (°C)			0.9359**	−0.6450	−0.8785**	0.9754**	−0.0492
Min. Temp (°C)				−0.8040*	−0.8190*	0.9794**	−0.2759
Rainfall (mm)					0.5619	−0.7101*	0.4739
Rainy day						−0.8192*	−0.0907
Sunshine (hr)							−0.1369

^*^P < 0.05, **P < 0.01.

### Evaluation of the genotypes

**Figure 2:**
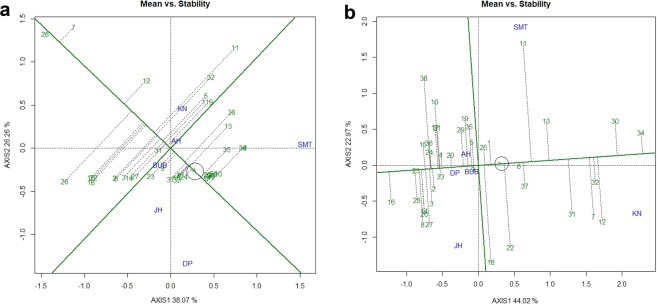
(**a**) “Mean vs. Stability” view of the GGE biplot considering gall index of 38 mungbean genotypes against *M*. *incognita* incidence across 6 test locations. (**b**) “Mean vs. Stability” view of the GGE biplot considering reproduction factor of 38 mungbean genotypes against *M*. *incognita* incidence across 6 testing locations. There was no transformation of data (transform = 0), and data were centred by means of the environments (centring = 2). The biplot was based on ‘row metric preserving’. Numbers correspond to genotypes as listed in Table 2. Locations are: SMT, Samastipur; KN, Kanpur; BUB, Bhubaneswar; AH, Aligarh; DP Durgapura; JH, Jorhat.

**Figure 3:**
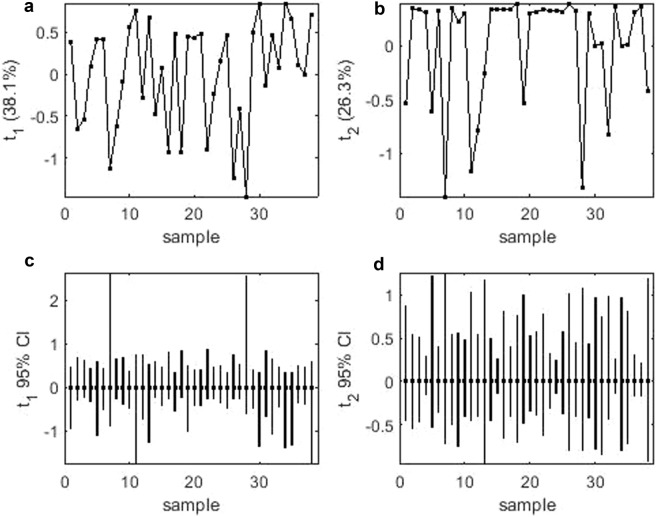
(**a**) PCA score values on PC1 versus genotype based on gall index; (**b**) PCA score values on PC2 versus genotype based on gall index; (**c**) PC1 score-values 95% BCa CLs (B = 1520),shown centered on nominal score-values; and (**d**) PC2 score-values 95% BCa CLs (B = 1520), shown centered on nominal score-values. Numbers correspond to genotypes as listed in Table 2.

**Figure 4:**
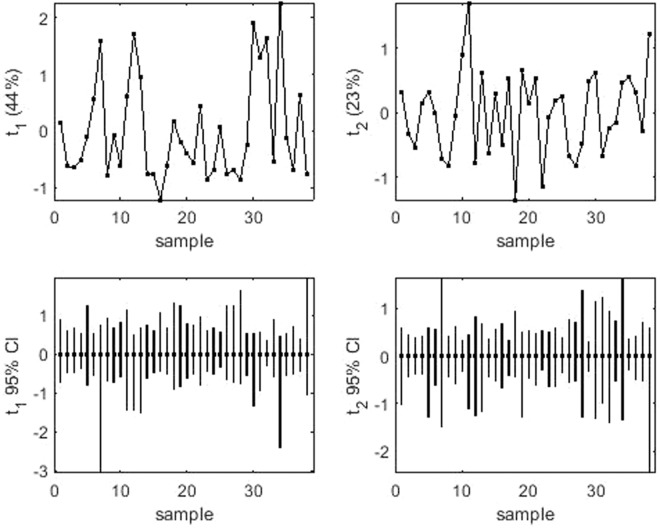
(**a**) PCA score values on PC1 versus genotype based on reproduction factor; (**b**) PCA score values on PC2 versus genotype based on reproduction factor; (**c**) PC1 score-values 95% BCa CLs (B = 1520),shown centered on nominal score-values; and (**d**) PC2 score-values 95% BCa CLs (B = 1520), shown centered on nominal score-values. Numbers correspond to genotypes as listed in Table 2.

Mean performance and stability of the genotypes across the locations were graphically portrayed through “Average Environment Coordination” (AEC) view of the biplot (Fig. 2). The environment-centered (centering = 2) and genotype-metric (SVP = 1) biplots considering gall index and reproduction factor were presented in Fig. 2a,b, respectively. The single arrow head line in the graph passing through biplot origin marked for “average environment” indicated higher disease severity thus reflected the poor performance of the genotypes. The “mean *vs*. stability” view of gall index, identified less gall scoring genotypes such as PM-10-12 (28), AKM-8802 (7), NVL-641 (26), IGKM-05-26-3 (12), IPM-410-3 (16) and AKM-4 (2) as resistant to moderately resistant genotypes (Fig. 2a). Conversely, considering reproduction factor, genotypes *viz*. IPM-410-3 (16), PM-10-12 (28), ML-2056 (23), IPM-2-3 (14), NVL-641 (26) and GM-04-02 (8) were recorded as resistant to moderately resistant genotypes. In the biplot, the greater projection of the genotypes from the “AEC abscissa” represented its less stability and vice versa. PM-10-12 (28) and ML-2056 (23) were detected as the most stable genotypes having short projection from “AEC abscissa” for the gall index and reproduction factor, respectively. Overall, considering both the parameters (gall index and reproduction factor) PM-10-12 (28) was the most ‘ideal’ genotype exhibited less root gall scoring and poor reproduction factor (moderately resistant) as well as high stability. Besides, the genotypes which were placed in proximity to the ‘ideal’ genotypes were considered as desirable. Therefore, NVL-641 (26) and IPM-410-3 (16) were detected as desirable genotypes with less gall scoring, poor reproduction factor and almost consistent performance. Further, considering the CI at 95% level regarding the individual genotypic scores on gall index and reproduction factors as well as environmental scores corresponding to PC1 and PC2 (Supplementary Table 2 and 3), computed through bootstrapping revealed that PC2 contributed more towards the detectable differences among the genotypes as reflected in the biplot (Figs. 3 and 4). Regarding gall index and reproduction factor it was affirmed that the ideal genotype PM-10-12 (28) was statistically different on the basis of PC2 scores of both gall index (Lower limit: −5.51 and Upper limit: 1.25) and reproduction factor (Lower limit: −3.22 and Upper limit: 1.48) from the two desirables genotypes *viz*. IPM-410-3 (16) and NVL-641 (26). On contrary, the two desirable genotypes did not exhibit significant differences corresponding to their PC2 scores concerning both the parameters.

### Evaluation of the test locations

**Figure 5:**
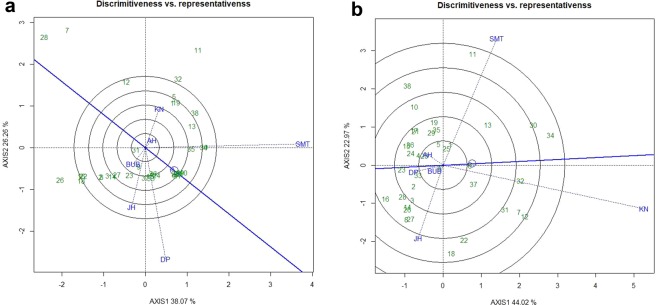
(**a**) “Discrimitiveness vs. Representativeness” view of GGE biplot based on gall index of *M. incognita* in 38 mungbean genotypes across 6 testing locations. (**b**) “Discrimitiveness vs. Representativeness” view of GGE biplot based on reproduction factor of *M. incognita* in 38 mungbean genotypes across 6 testing locations. There was no transformation of data (transform = 0), anddata were centred by means of the environments (centring = 2). The biplot was based on ‘row metric preserving’. Numbers correspond to genotypes as listed in Table 2. Locations are: SMT, Samastipur; KN, Kanpur; BUB, Bhubaneswar; AH, Aligarh; DP Durgapura; JH, Jorhat.

The relationship among the test locations and identification of ideal test location is an important attribute of GGE biplot. The first two principal components (PCs) explained 64.33% and 66.99% of the total variation of environment-centered gall scoring and reproduction factor data of *M. incognita*, respectively. In case of gall index, PC1 (*M. incognita* incidence) and PC2 (resistance stability) accounted for 38.07% and 26.26% of total variation, respectively (Fig. 5a) whereas, for reproduction factor, the PC1 (*M. incognita* incidence) and PC2 (resistance stability) accounted for 44.02% and 22.97% of total variation, respectively (Fig. 5b). All test locations were connected through environment vectors and it was depicted that in case of gall index (Fig. 5a) Kanpur and Aligarh; Aligarh and Samastipur as well as Bhubaneswar, Jorhat and Durgapura exhibited acute angle indicating positive correlation and close relationship with each other. On the contrary, obtuse angle was exhibited between Samastipur and Kanpur as well in between Samastipur, Kanpur with other three locations except Aligarh revealing a paradoxical relationship between these locations. However, this relationship was not consistent for reproduction factor. In case of reproduction factor, acute angle was observed between Jorhat and Bubhaneswar, between Bhubaneswar, Durgapura and Aligarh. Contrarily, Kanpur and Samastipur locations exhibited obtuse angle with all locations (Fig. 5b). Similarly, Jorhat, Aligarh and Durgapura also exhibited obtuse or nearly obtuse angle with each other. While, Bhubaneswar and Durgapura demonstrated almost similar positions indicating analogous genotypic response towards *M. incognita*, thus one of the locations could be curtailed down in the future testing program.

In GGE biplot approach, three parameters *viz*., ‘discrimination’ power (ability to segregate the tested genotypes), ‘representativeness’ (ability to represent the mega environment) and “desirability index” were usually deliberated for test locations evaluation. In the present study, it was detected that among the test locations considering gall scoring Samastipur exhibited longest environmental vectors thus identified as most “discriminating location” having the power of genotypes discrimination amid other locations. Additionally, Samastipur exhibited smallest angle with “AEC abscissa”, thus identified as most ‘Representative’ test locations. On the contrary, in case of reproduction factor, Kanpur reflected longest environmental vectors as well as smallest angle with AEC abscissa” therefore, considered as discriminating as well as most representative test location. The “desirability index” of testing location is the cumulative effect of both ‘discriminativeness’ of a location and ‘representativeness’. Finally, Samastipur location with highest desirability index (Table 4) was considered as ‘ideal’ testing location for root-knot nematode screening. In addition, Kanpur could be entitled as ‘supplementary’ testing location. These two locations would be ideal for selecting genotypes with general adaptability.

**Table 4:** Standardized test locations evaluation parameters.

Location	Discrimitivenes	Representativeness	Desirability Index
SMT	1.383	0.575	0.724
KN	1.260	0.482	0.667
BUB	0.276	0.235	0.425
AH	0.232	0.212	0.049
DP	1.095	0.295	0.323
JH	0.821	0.392	0.322

### Identification of environment-specific genotypes and mega-environment delineation

**Figure 6:**
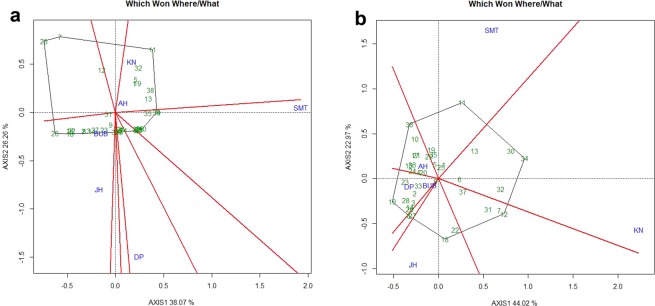
(**a**) “Which-won-where” view of the GGE biplot based on gall index of *M. incognita* in 38 mungbean genotypes across 6 testing locations. (**b**) “Which-won-where” view of the GGE biplot based on reproduction factor of *M. incognita* in 38 mungbean genotypes across 6 testing locations. There was no transformation of data (transform = 0), and data were centred by means of the environments (centring = 2). The biplot was based on ‘row metric preserving’. Numbers correspond to genotypes as listed in Table 2. Locations are: SMT, Samastipur; KN, Kanpur; BUB, Bhubaneswar; AH, Aligarh; DP Durgapura; JH, Jorhat.

Two-dimensional polygon view, “which-won-where” graph of GGE biplot was developed to identify genotype for a specific test environment (Fig. 6). Genotypes at the vertices of the polygon are either the best or the poorest performer in the environment falling within the sectors and contributed maximum in interactions^34,35^. In present study it was observed that among two “which-won-where” graphs (Fig. 6a,b), the biplot considering reproduction factor (Fig. 6b) with well distributed polygon was the most informative for discriminating environments efficiently. The equality lines partitioned the six locations into four mega environments based on gall index (Fig. 6a). Samastipur and Durgapura both represented two different mega environments (ME-I and ME-II). The third mega environment (ME-III) was constituted by Aligarh and Kanpur where Aligarh can be dropped while Bhubaneswar and Jorhat included in fourth mega environment where Jorhat can be eliminated for future testing based on desirability index (ME-IV). Similarly, in the case of reproduction factor, four “Mega Environments” were also detected. Kanpur (ME-I), Samastipur (ME-II) and Aligarh (ME-III) individually depicted as three different mega environments, whereas, Durgapura, Bhubaneswar and Jorhat comes under the fourth mega environment (ME-IV) where except Bhubaneswar the rest two testing locations can be eliminated. Finally, considering both the factors, all the testing locations could be partitioned into four different mega environments. Based on both biplots genotypes such as NVL-641 (26), IPM-410-3 (16), GGG-10-14 (3) and AKM-4 (2) were located in the downstream of the AEC abscissa and in the vertex which indicated that these genotypes were the promising against *M. incognita*.

### Biochemical characterization of resistant and susceptible genotypes

The total phenol content of both susceptible and resistant mungbean genotypes ranged between 140.69 to 209.66 GAE mg/100 g seed (Table 5). The highest total phenol content was noticed in resistant genotypes *viz*., NVL 641 and PM 10-12. Ascorbic acid content was highest in PM-10-12 and it was lowest in susceptible genotype (IPM 9901-8). The PAL activity was higher in resistant genotypes from 0.153 to 0.161 µmol *trans-*cinnamic acid formed/mg of protein/ hour. The PPO activity was lowest in susceptible genotype IPM 9901-8 (137.75 Units/mg protein).

**Table 5:** Biochemical characterization of resistant and susceptible genotypes of mungbean against *M. incognita* on the basis of total phenols, ascorbic acid, PAL and PPO activity.

Genotype	Total phenols	Ascorbic acid	PAL	PPO
IPM 410-3	140.69 ± 4.97	9.03 ± 0.32	0.161 ± 0.011	215.56 ± 3.4
IPM-9901-8	151.26 ± 4.21	7.91 ± 0.58	0.126 ± 0.032	137.75 ± 3.9
NVL- 641	209.66 ± 9.66	9.31 ± 0.58	0.153 ± 0.013	194.70 ± 5.2
PM-10-12	205.98 ± 5.74	10.15 ± 0.16	0.156 ± 0.023	190.89 ± 4.7
PUSA-1371	160.92 ± 2.87	9.59 ± 0.32	0.140 ± 0.009	203.64 ± 3.9
PUSA-1472	159.08 ± 7.60	8.29 ± 0.32	0.158 ± 0.044	154.07 ± 4.9
PUSA-1471	198.16 ± 5.57	8.01 ± 0.43	0.153 ± 0.003	155.88 ± 3.8

## Discussion

Host-plant resistance towards root-knot nematode infestation is an effective approach in mungbean for reducing yield penalties caused due to the presence of nematode population beyond the economic threshold level (ETL). Unfortunately, screening of genotypes for disease resistance has the drawback of the irregularity of environmental conditions and pest population^1,29,35,36^. Likewise, genotypes screening against *M. incognita* is a complicated task due to the complex behavioural pattern of the nematode with ambiguous symptoms of gall formation^21^. Large scale screening of diverse genotypes in varied environments against nematode infestation is costly which create the necessity of elucidation of ideal test environments in terms of excellent genotype discrimination power as well as identification of the mega environments with different cross over interactions. “Mega-environment” was defined as a portion of the growing region which is homogenous enough to lead genotypes to perform similarly^37^, or as a group of geographical locations where the same or similar cultivars performed the best across years^38^. Presence of mega environments with winning genotypes in each environment suggested genotypes with specific adaptation in respective mega environment^34,37,39–41^. Therefore, GGE biplot analysis provides an easy and comprehensive solution to genotype by environment data analysis, for effective evaluation of the genotypes as well as identification of the target test locations^42,43^.

In the present study, a panel of 38 promising genotypes of mungbean tested over six diverse locations exhibited significant genotype x environment interaction towards *M. incognita* infestation. ANOVA validated significant differences among the genotype, environment as well as genotype-environment interaction. Further, mean performance over the locations also confirmed inconsistent performance of tested genotypes towards *M*. *incognita* infestation which may be due to variation in weather parameters, soil types as well as diverse ecologies of the tested environments that could have a significant role towards increment of *M*. *incognita* population and further reproduction as well as survival. Existence of significant genotype by environment interaction (GEI) towards *M*. *incognita* infestation rationalized elucidation of genotypic performance in multiple locations before drawing valid recommendation regarding genotypes ranking. Concerning environment variables, it was observed that rainfall and minimum temperature were detected as most influential factors for determining gall scoring, population and reproduction factor towards *M*. *incognita* invasion. Unpredictable weather parameters along with GE interaction are decisive factors in crop breeding^44^, which justify genotype evaluation in multi-environment contemplating genotypic performance and stability^45,46^.

In GGE biplot analysis, the complex GEI are simplified in different PCs and presented graphically against various PCs. In the present study, for both the characters i.e. gall index and reproductive factors, the first two PCs explained more than 60% of the total variation confirming adequate representation of the variability for root-knot nematode infestation. Differential responses of tested genotypes to diverse environments due to the presence of cross over interactions were depicted in the “mean vs. stability view” of the biplot for both the characters, where genotype ranking changed from one environment to another. Previous reports stated the importance of crossover interaction in breeding programme and strongly recommended the implication of breeding for specific adaptation^34,47–49^. It was observed that among the tested genotypes, though GM-04-02 (8) exhibited good performance against *M*. *incognita*, however, PM-10-12 (28) was detected as ‘ideal’ genotype with most stable performance along with the lowest projection onto “AEC ordinate” for both the parameters. Additionally, IPM-410-3 (16) and NVL-641 (26) with consistent performance and less infestation by *M*. *incognita* placed closer to the ideal genotype and “AEC abscissa”, thus were depicted as ‘desirable’ genotypes have varying capacity to respond against pest infestation by inducing a long-lasting, broad-spectrum, systemic resistance^50^. Moreover, these genotypes having diverse ability and mechanism to perceive environmental signals into their developmental pathways for generating wide range of adaptive capacities over time that provide varied response towards biotic stresses^51^. The ‘ideal’ and ‘desirable’ genotypes identified in the present study with durable resistance could be a valuable resource for mungbean resistance breeding programme against root-knot nematode infestation. This approach has been successfully deployed for identifying resistant genotypes in different crops against the different pathogen^1,28,29,31,52^.

An important feature of GGE biplot is to identify an optimum number of test locations to maximize the trait heritability and genetic gain in minimum trial cost^53^. In the present study considering both the parameters Bhubaneswar and Jorhat resembled homology regarding root-knot nematode infestation. The positive relationship between the test environments justified dropping of Jorhat location with similar information. Presence of a close relationship among the test locations indicated the existence of non-crossover GEI, where genotypes performance was consistent. Presence of both crossover and non-crossover GEI was reported earlier^41,49,54^. Positive correlation among the testing locations was not in accordance with their agro-ecological zones. Weather parameters and soil types are the two principal factors of an environment determining genotypic performance in diverse locations^41^. Finally, it was observed that rainfall and minimum temperature were the most influential factors and play an instrumental role in *M*. *incognita* invasion. Through GGE biplot approach, all the tested locations could be grouped into four distinct “mega-environments”, which confirmed the presence of cross-over type of GEI in the present experiment. Furthermore, GGE biplot identified Samastipur and Kanpur as the ‘ideal’ and ‘supplementary’ testing sites, respectively, for screening of mungbean genotypes against root-knot nematode. Besides, among the tested genotypes, PM-10-12 (28) was recognized as ‘ideal’ genotype with most stable performance against *M*. *incognita* infestation. Further, the resistant and susceptible genotypes were characterized on the basis of enzymatic and antioxidant activities. The total phenols, ascorbic acid, PAL and PPO activity were higher in resistant genotypes as compared with susceptible genotypes. The results were supported by the previous findings where it was reported that the stress related protective enzymes play key role in scavenging free radicles and reactive oxygen species towards triggering incompatible reaction against root-knot nematodes^55–57^. These biochemical markers also helped in identification of root-knot nematode resistant genotypes in mungbean. The judicious utilization of identified genotypes holds great promise in future resistance breeding programmes in mungbean.

## Materials and Methods

### Initial testing

In preliminary screening, field evaluation of 250 germplasm of mungbean was carried out during the year 2013 and 2014 in root-knot nematode sick micro plots of New Research Farm of Indian Institute of Pulses Research (IIPR), Kanpur, India located at 26°27′N latitude, 80°14′E longitude and 152.4 above msl. Root-knot nematode population was multiplied in the naturally infested micro plots by growing pigeonpea for two years and then blackgram during wet season followed by chickpea during winter season. Soil of the micro plots was sandy loam with 7.5 pH, from which 200 cc soil samples were drawn with the help of soil auger. *M*. *incognita* from soil sample were extracted using Cobb’s wet sieving and sedimentation technique for estimating total nematode population in the soil^58^. At the time of sowing, the average nematode population level was 178 juveniles per 200 cc soil. After growing host crop, the nematode population was estimated and inoculum was added to those microplots where the initial population was less. Before scoring, the nematode population of each microplot was calibrated for at least 178 juveniles/200 cc soil. The tested genotypes were sown in a single row of 2 m length. After 45 days, four plants of each genotype were removed from the soil along with their root systems. The root system was washed gently under tap water and observations were recorded for each plant on gall index (GI) based on a 1–5 scale as: 1 = no galls/egg masses: Highly resistant; 2 = 1–10 galls/egg masses: Resistant; 3 = 11–30 galls/egg masses: Moderately resistant; 4 = 31–100 galls/egg masses: Susceptible and 5 = >100 galls/egg masses: Highly Susceptible^59^.

### Preparation of nematode inoculum for individual location

**Figure 7:**
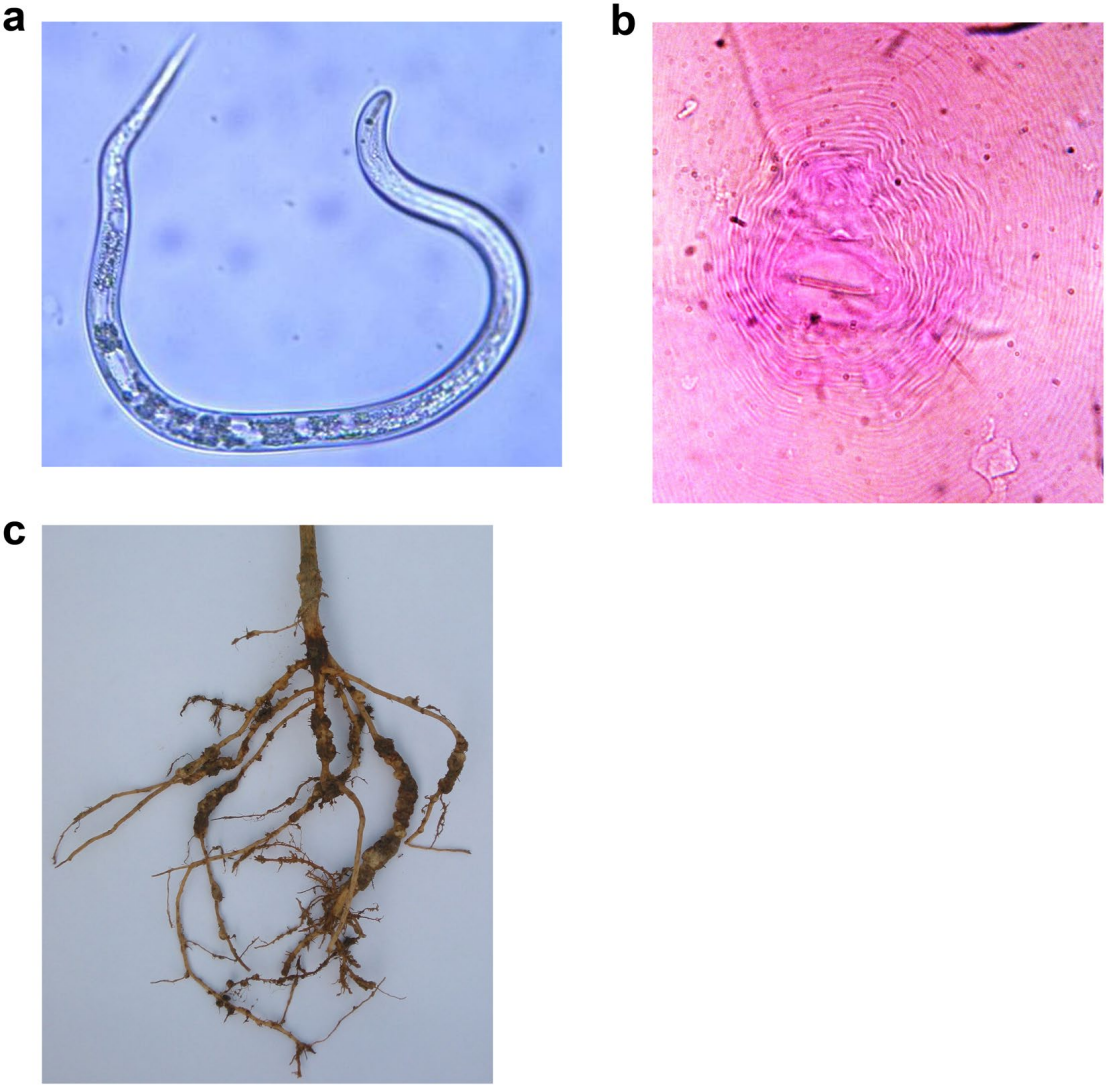
Root knot nematode, *M. incognita* and its host-plant reaction. (**a**) The infective stage of *M. incognita*. (**b**) Perineal pattern of *M. incognita*. (**c**) Presence of gall in the infected plants.

The infective stage of *M. incognita* (Fig. 7a) was taken from culture pots maintained at respective centres. The identity of species *M*. *incognita* was confirmed based on the perineal pattern (Fig. 7b) at all locations. *M*. *incognita* was mass cultured from a single egg mass on a susceptible cultivar maintained in greenhouse at 25 °C ± 2. The infested plants (Fig. 7c) were uprooted from the pots, washed with tap water, cut into smaller pieces, and vigorously shaken in a flask containing 0.5% NaOCl for 5 min and the egg masses were collected in 51 mm diameter petri-plates and kept in incubator for hatching of 2^nd^ stage juveniles^60^. These freshly hatched juveniles were used in the screening of the mungbean genotypes at different locations for validation in multi-environment evaluation.

### Recording of observations on galls, gall index and reproduction factor on different genotypes

Root-knot nematodes infestation is characterized based on two parameters; first, formation of galls in the root due to hyperplasia and hypertrophy of the cells, second, reproduction factor which is calculated based on the final population and the initial population. Many times the nematodes penetrate the root and form galls, but majority of the nematodes may fail to mature and reproduce, resulting in low reproduction factor in spite of high gall index. Therefore, both gall index and reproduction factor are important to determine the susceptible or resistant reaction of genotypes^61^. Therefore, for data recording steam sterilised field soil was filled in 6” diameter earthen pots and a total of 38 genotypes of mungbean were sown in pots at all the test locations during middle of July to 1^st^ week of August. Four seeds of each genotype were sown in respective pots maintaining five replications. Three plants were maintained in each pot after germination. At two leaf-stage, 200 freshly hatched 2^nd^ stage juveniles of *M. incognita* were added with the help of pipette in the root zone of the plants in pots. The pots were watered whenever it was required. After completion of one biological cycle of nematode after 30 days, the plants were removed only after 45 days and observations were recorded on the number of galls and gall index was calculated based on a 1–5 scale^59^. As the crop matures in 60–70 days and roots starts decaying subsequently at maturity, counting galls and egg masses at this stage becomes impossible. Pot soil was processed to extract J2 of *M. incognita* as per modified Cobb’s decanting and sieving technique^62^. Root population was counted taking 2 g of roots and staining in acid fuschin and pressing in two slides under stereoscope microscope. Final population was calculated by adding soil and root population. The host status was determined by measuring the reproductive capacity of the nematode using the following formula^61^: R = Pf/Pi; where R is reproduction factor, Pf represents the final population of juveniles and eggs recovered from the soil and roots of infected plants, Pi the initial population^63^.

### Multi-environment evaluation

After initial screening finally, 38 mungbean genotypes comprised of advance breeding lines and released varieties which were resistant or moderately resistant against root-knot nematodes were tested using sick plots for *M*. *incognita* in three replications across six diverse locations in India during wet season of 2016–17. Information regarding plant materials utilized in the present study were presented in Supplementary Table 1. Test locations were decisively selected based on prevalence of root-knot nematode problem. Further, theses locations represent wide diversity of agro-climatic conditions (Table 6).

**Table 6:** Description of test locations in India.

Sl No.	Location	Biplot Code	Elevation (msl)	Latitude & longitude	Soil Type	Avg. rainfall (mm)	RH (%)	Temp (°C)	
								Min	Max
1	Dr. Rajendra Prasad Central Agricultural University, **Samastipur**, Bihar	SMT	52	25°90′N 85°60′E	Loam	60.8	81.70	26.1	32.4
2	Chandra Sekhar Azad University of Agriculture & Technology, **Kanpur**, Uttar Pradesh	KN	126	26°12′N, 80°71′E	Loam	90.4	75.85	25.9	32.8
3	Orissa University of Agriculture & Technology, **Bhubaneshwar**, Orissa	BUB	45	17′ 31′′-20'31′′N 81'31′′-87'30′′E	Sandy Loam	361.9	90.25	26.0	32.0
4	Aligarh Muslim University, **Aligarh**, Uttar Pradesh	AH	178	28 °35′N 78 °82′E	Sandy Loam	87.6	61.39	25.1	32.7
5	Rajasthan Agricultural Research Institute, **Durgapura**, Rajasthan	DP	390	26°51′N 75°47′E	Sandy	175.3	65.50	24.9	34.0
6	Assam Agriculture University, **Jorhat**, Assam	JH	94	26°28′N 94°65′E	Sandy Loam	635.6	86.0	25.3	32.8

### Construction of GGE Biplot

The GGE biplot is a graphical representation that displays genotype (G) and genotype by environment (GE) interaction, for determination of magnitude and nature of GE interaction in multi-location data set^64^. This biplot is constructed through plotting first the principal component (PC1) scores of the genotypes and the environments against their respective scores for the second principal component (PC2) that result from “singular value decomposition” (SVD) of environment-centered data by estimating each element of the matrix using the following formula:

$${Y}_{ij}=\mu +{e}_{j}+\mathop{\sum }\limits_{n=1}^{N}{\lambda }_{n}{\gamma }_{in}{\delta }_{jn}+{\varepsilon }_{ij}$$

where,

Y_**ij**_ = mean incidence of i^th^ genotype (i = 1, …, I) in the j^th^ environment (j = 1, .., J)

µ = grand mean

e_**j**_ = environment deviations from the grand mean

λ_n_ = The eigen value of PC analysis axis

γ_in_ and δ_jn_ = genotype and environment principal components scores for axis n

N = number of principal components retained in the model and

ε_ij_ = Residual effect **~ N** (0, σ^2^)

The present data on the genotypic response towards *M*. *incognita* infestation across the locations were analyzed without scaling (‘Scaling 0’ option) to generate a tester centred (centring 2) GGE biplot^34^. Appraisal of genotypic performance and stability was executed through “average environment coordination” (AEC) view of the GGE biplot which facilitated genotype comparison based on mean of disease score and stability across environments within a mega-environment^35^. The axis of the “AEC abscissa”, represented as a single-arrowed line indicated higher mean performance of the genotypes in terms of gall scoring of *M. incognita* i.e., higher susceptibility, whereas double arrowed line perpendicular to “AEC abscissa” passing through biplot origin, known as “AEC ordinate” represented a decrease in stability of genotype^23^. Likewise, test location evaluation was carried out through “discriminating power vs. representativeness” view of the GGE biplot, where the ‘ideal’ test environment should have both discriminating ability for genotypes and representative of the mega-environment^31^. Additionally, “desirability index” of the test locations has been enumerated considering association among the test environments and distance from the ideal genotype, based on the “AEC considering genotypic stability and adaptability^64^. Furthermore, the grouping of test environments with the similar response into the different mega environments was visualized through the “which-won-where” view of the GGE biplot^37^. Additionally, for improving precision of GGE biplot, CI at 95% level was computed through bootstrapping for drawing statistical inference about individual principal component scores of both, genotypes and environments as suggested earlier^62^. During CI calculation test locations were considered as columns (p = 6) whereas, genotypes were treated as rows (n = 38). Re-sampling was carried out 40 times to the number of rows (B = 1520) for generating an empirical sampling distributing.

### Biochemical characterization

#### Total phenol

Total phenols were extracted from 0.5 g defatted mungbean flour from each genotype using 5 ml of 70% ethanol centrifuged at 3000 g for 15 min. The supernatant was used for determining the total phenol content using the Folin-Ciocalteau method with slight modifications as suggested by earlier worker^65^. A standard curve of gallic acid was simultaneously prepared. The calibration curve was linear in the range 25 to100 µg/mL. Results were expressed in terms of gallic acid equivalent (GAE) mg/100 g seed.

#### Ascorbic acid

The ascorbic acid was measured by Titrimetric method^66^. The amount of ascorbic acid was calculated by using formulae as follows:

$$Amount\,of\,Ascorbic\,acid(mg\,per\,100\,g)=\frac{{\rm{X}}({\rm{mg}})\,\times \,V2\,\times \,Z}{{\rm{V}}1\,\times \,{\rm{Y}}\,\times \,{\rm{Weight}}\,{\rm{of}}\,{\rm{sample}}\,({\rm{g}})}\,\times \,100$$

where,

X = mg of standard ascorbic acid.

V_1_ = Titre value of standard ascorbic acid against dye.

V_2_ = Titre value of sample against dye.

Y = Amount of aliquot taken (ml) for estimation.

Z = Total volume made up of extracted sample.

### PAL (Phenylalanine ammonia lyase) activity

The PAL estimation was done as per the standard protocol^67^ and expressed in terms of µmole of transcinnamic acid formed/mg protein/hr. The amount of *trans*-cinnamic acid formed was measured using the molar extinction coefficient of standard *trans*-cinnamic acid (19730) (As per Sigma aldrich protocol). The reaction rate was calculated as micromole *trans-*cinnamic acid formed per mg of protein per hour.

### PPO (Polyphenol oxidase) activity

PPO activity was assessed as per the Sigma Aldrich protocol and expressed as units/mg protein. A sample of 0.5 g of overnight soaked seeds was grinded into 5 ml of 50 mM Potassium phosphate buffer, pH 6.5 containing 5 mM mercaptoethanol followed by centrifugation @ 12000 g for 20 min. The supernatant was used as enzyme source.2.6 ml of 50 mM potassium phosphate buffer, pH 6.5 was taken in cuvette followed by addition of 100 μl of each 5 mM of L-DOPA, 2.1 mM of Ascorbic acid and 0.065 mM of EDTA. The reaction was initiated by addition of 100 μl of enzyme extract. The decrease in the absorbance was monitored at 265 nm for 3 minute at 30 second interval.

Calculations:

$$\frac{Units}{mg\,protein}=\frac{(\frac{{\rm{A}}265\,{\rm{nm}}}{{\rm{\min }}}\,{\rm{Test}}\,-\,\frac{{\rm{A}}265\,{\rm{nm}}}{{\rm{\min }}}{\rm{Blank}})}{(0.001)\times mg\,protein}$$

where,

0.01 = the change in A265 nm/minute per unit polyphenol oxidase at pH = 6.5

### Data analysis

The effects of environments, genotype and their interactions were determined by analysis of variance (ANOVA), using mixed-model analysis in GENSTAT (trial version 18; VSN International, Hemel Hempstead, UK). The ANOVA explained the partition of variation due to the effect of genotypes, environment and their interaction. To identify the relationship between weather variables and *M*. *incognita* infestation, Spearman’s correlation coefficient was calculated. The GGE biplot analysis was done considering two parameters *viz*. gall scoring and reproduction factor, for determining the intensity of *M*. *incognita* infestation in the genotypes by using the R software (R Development Core Team, Vienna).

## Supplementary information

Supplementary information.
