# Supplementary material for: Delineation of Genotype-by-Environment interactions for identification and validation of resistant genotypes in mungbean to root-knot nematode (Meloidogyne incognita) using GGE biplot
Source: Sci Rep. 2020 Mar 5;10:4108. doi: 10.1038/s41598-020-60820-x (PMC7058080; doi:10.1038/s41598-020-60820-x)
Supplement: Supplementary file 1 — Supplementary information. [file 41598_2020_60820_MOESM1_ESM.doc]

**Supplementary Table 1:** Information regarding the mungbean genotypes

| **Sl No.** | **Genotype** | **Pedigree** | **Developing Centre** | **Days to 50 % flowering** | **Days to maturity** | **Yield**  **(kg/ha)** |
| --- | --- | --- | --- | --- | --- | --- |
| 1 | AKM -12-10 | AKM 9911 x BM 2003-2 | **Dr. Punjab Rao Deshmukh Krishi Vidyapeethth**, Akola, Maharashtra, India | 35 | 64 | 876 |
| 2 | AKM-4 | BM 4 x PS 7 | **Dr. Punjab Rao Deshmukh Krishi Vidyapeethth**, Akola, Maharashtra, India | 40 | 68 | 1114 |
| 3 | AKM-8802 | MH 1 x PIMS 4 | **Dr. Punjab Rao Deshmukh Krishi Vidyapeethth**, Akola, Maharashtra, India | 38 | 65 | 817 |
| 4 | DGG-3 | GG4 x TM 98-50 | **University of Agricultural Science, Dharwad**, Karnataka, India | 38 | 65 | 630 |
| 5 | DGG-5 | Mutant of LGG 460 | **University of Agricultural Science, Dharwad**, Karnataka, India | 37 | 78 | 828 |
| 6 | DGG-6 | SM x TM 96-2 | **University of Agricultural Science, Dharwad**, Karnataka, India | 35 | 64 | 944 |
| 7 | GGG- 10-14 | ? | **Agricultural Research Station**, Gujrat, India |  |  |  |
| 8 | GM -04-02 | GM 9303 x PB 1 | **Sardarkrushinagar Dantiwada University**, Gujrat, India | 34 | 72 | 733 |
| 9 | GM- 11-02 | GM 9926 x Pusa Vishal | **Sardarkrushinagar Dantiwada University**, Gujrat, India | 39 | 67 | 866 |
| 10 | HUM-1 | BHUM1 x Pant U30 | **Banaras Hindu University**, Varanasi, Uttar Pradesh, India | 37 | 70 | 790 |
| 11 | HUM-27 | Selection from HUM 12 | **Banaras Hindu University**, Varanasi, Uttar Pradesh, India | 37 | 64 | 926 |
| 12 | IGKM- 05-26-3 | Malviya Jyoti x TM 99-2 | **Indira Gandhi Krishi Viswavidyalaya**, Raipur, Chattishgarh, India | 35 | 65 | 889 |
| 13 | IPM- 2-3 | IPM 99-125 x Pusa Bold 2 | **ICAR-Indian Institute of Pulses Research**, Kanpur, Uttar Pradesh, India | 36 | 62 | 958 |
| 14 | IPM -2-14 | IPM 99-125 x Pusa Bold 2 | **ICAR-Indian Institute of Pulses Research**, Kanpur, Uttar Pradesh, India | 36 | 69 | 873 |
| 15 | IPM- 2K15-4 | EC 398897 x IPM 99-125 | **ICAR-Indian Institute of Pulses Research**, Kanpur, Uttar Pradesh, India | 39 | 69 | 761 |
| 16 | IPM -410-3 | IPM 03-1 x NM 1 | **ICAR-Indian Institute of Pulses Research**, Kanpur, Uttar Pradesh, India | 36 | 67 | 1068 |
| 17 | IPM 9901-6 | IPM 99-125 x Pusa Bold 2 | **ICAR-Indian Institute of Pulses Research**, Kanpur, Uttar Pradesh, India | 36 | 66 | 1088 |
| 18 | IPM -9901-8 | IPM 99-125 x Pusa Bold 2 | **ICAR-Indian Institute of Pulses Research**, Kanpur, Uttar Pradesh, India | 35 | 64 | 903 |
| 19 | KM-2342 | KM 132 x KM 2241 | **Chandra Sekhar Azad University of Agriculture & Technology**, Kanpur, Uttar Pradesh, India | 38 | 63 | 951 |
| 20 | MH-275 | PDM 116 x Gujarat 1 | **CCS Haryana Agricultural University**, Hisar, Haryana, India | 39 | 67 | 742 |
| 21 | MH-810 | MH 96-1 x 2KM 114 | **CCS Haryana Agricultural University**, Hisar, Haryana, India | 34 | 62 | 1045 |
| 22 | MH-934 | MH 96-1 x 2KM 117 | **CCS Haryana Agricultural University**, Hisar, Haryana, India | 36 | 64 | 971 |
| 23 | ML-2056 | ML 1165 x ML 1191 | **Punjab Agricultural University**, Ludhiana, Punjab, India | 39 | 66 | 891 |
| 24 | ML-2333 | ML 1349 x VC 6370 A | **Punjab Agricultural University**, Ludhiana, Punjab, India | 35 | 63 | 995 |
| 25 | NVL-516 | NVL 550 x NVL 618 | **Nirmal Seeds Pvt. Ltd**., Jalgaon, Maharashtra, India | 38 | 74 | 1011 |
| 26 | NVL-641 | NVL 605 x NVL 501 | **Nirmal Seeds Pvt. Ltd**., Jalgaon, Maharashtra, India | 37 | 64 | 666 |
| 27 | PM- 09-11 | PM 5 x Bina Mung | **Govind Ballabh Pant University of Agriculture and Technology**, Pantnagar, Uttarakhand, India | 38 | 67 | 772 |
| 28 | PM-10-12 | PM 3 x UPM 99-3 | **Govind Ballabh Pant University of Agriculture and Technology**, Pantnagar, Uttarakhand, India | 42 | 69 | 939 |
| 29 | PUSA-0672 | 11/395 x ML 267 | **ICAR-Indian Agricultural Research Institute**, New Delhi, India | 38 | 75 | 680 |
| 30 | PUSA-1371 | Pusa Vishal x IPM 02-19 | **ICAR-Indian Agricultural Research Institute**, New Delhi, India | 36 | 76 | 772 |
| 31 | PUSA-1471 | - | **ICAR-Indian Agricultural Research Institute**, New Delhi, India | 36 | 64 | 958 |
| 32 | PUSA-1472 | - | **ICAR-Indian Agricultural Research Institute**, New Delhi, India | 36 | 64 | 1052 |
| 33 | RMG-1028 | MUM 2 x RMG 62 | **Rajasthan Agricultural Research Institute,** Durgapura, Rajasthan India | 38 | 78 | 840 |
| 34 | RMG-1030 | RMG 275 x RMG 492 | **Rajasthan Agricultural Research Institute,** Durgapura, Rajasthan India | 35 | 64 | 920 |
| 35 | SGC-20 | AAU 34 x T 44 | **RRS, Assam Agricultural University**, Shilongani, Assam, India | 37 | 66 | 1044 |
| 36 | TARM-1 | RUM 5 x TPM 1 | **Bhaba Atomic Research Centre**, Trombay, Maharashtra, India | 37 | 68 | 1170 |
| 37 | TMB-45 | TM 96-2 x VC 6379 | **Bhaba Atomic Research Centre**, Trombay, Maharashtra, India | 38 | 74 | 628 |
| 38 | VGG 05-006 | VRM 1 (Gg) x Vellore local | **National Pulses Research Centre**, Tamil Nadu Agricultural University, Vamban, Tamil Nadu, India | 39 | 68 | 784 |

**Supplementary Table 2:** Deviations of **mean gall index** of 38 mungbean genotypes tested at six environments from the corresponding environmental means. Genotype and environment scores corresponding to the PC1 and PC2 and at CLs @ 95 % enumerated through bootstrapping

| Sl No. | **SMT** | **KN** | **BUB** | **AH** | **DP** | **JH** | **Mean** | **PC1** | **Lower limit** | **Upper limit** | **PC2** | **Lower limit** | **Upper limit** |
| --- | --- | --- | --- | --- | --- | --- | --- | --- | --- | --- | --- | --- | --- |
| 1 | 3.8 | 4.0 | 3.0 | 4.0 | 3.0 | 4.0 | 3.6 | 0.39 | 0.02 | 1.05 | -0.54 | -1.59 | -0.24 |
| 2 | 2.6 | 4.0 | 3.0 | 3.7 | 4.0 | 4.0 | 3.6 | -0.65 | -1.80 | -1.11 | 0.35 | -0.11 | 0.93 |
| 3 | 2.7 | 4.0 | 3.0 | 3.9 | 4.0 | 4.0 | 3.6 | -0.54 | -1.50 | -0.84 | 0.33 | -0.03 | 0.87 |
| 4 | 3.3 | 4.0 | 3.0 | 4.0 | 4.0 | 4.0 | 3.7 | 0.09 | -0.27 | 0.38 | 0.31 | 1.89 | 2.43 |
| 5 | 3.7 | 4.0 | 2.3 | 3.8 | 3.0 | 4.0 | 3.5 | 0.41 | -0.42 | 0.91 | -0.61 | -1.66 | 0.18 |
| 6 | 3.7 | 4.0 | 3.3 | 4.0 | 4.0 | 4.0 | 3.8 | 0.41 | 0.49 | 1.33 | 0.33 | 1.30 | 2.08 |
| 7 | 2.0 | 5.0 | 3.0 | 4.0 | 3.0 | 2.6 | 3.3 | -1.12 | -1.26 | 1.70 | -1.40 | -3.69 | -1.68 |
| 8 | 2.6 | 4.0 | 3.1 | 4.0 | 4.0 | 4.0 | 3.6 | -0.63 | -1.79 | -1.08 | 0.35 | 0.02 | 1.02 |
| 9 | 3.1 | 4.0 | 2.3 | 4.0 | 4.0 | 4.0 | 3.6 | -0.09 | -0.89 | -0.16 | 0.23 | -0.29 | 0.56 |
| 10 | 3.8 | 4.0 | 3.0 | 4.0 | 4.0 | 4.0 | 3.8 | 0.55 | 0.75 | 1.51 | 0.29 | 1.40 | 2.17 |
| 11 | 4.0 | 4.0 | 3.0 | 4.0 | 3.0 | 2.6 | 3.4 | 0.75 | -0.91 | 1.23 | -1.17 | -2.64 | -0.87 |
| 12 | 3.0 | 5.0 | 3.2 | 4.0 | 3.0 | 4.0 | 3.7 | -0.28 | -0.49 | 0.70 | -0.79 | -2.82 | -2.07 |
| 13 | 3.8 | 4.0 | 2.1 | 4.0 | 4.0 | 3.0 | 3.5 | 0.67 | -0.57 | 0.95 | -0.26 | -0.59 | 0.99 |
| 14 | 2.8 | 4.0 | 3.0 | 3.7 | 4.0 | 4.0 | 3.6 | -0.48 | -1.41 | -0.72 | 0.34 | 0.03 | 0.84 |
| 15 | 3.3 | 4.0 | 3.3 | 4.0 | 4.0 | 4.0 | 3.8 | 0.07 | -0.43 | 0.28 | 0.34 | 2.13 | 2.63 |
| 16 | 2.3 | 4.0 | 3.0 | 4.0 | 4.0 | 4.0 | 3.5 | -0.93 | -2.29 | -1.71 | 0.34 | -0.13 | 1.25 |
| 17 | 3.7 | 4.0 | 3.3 | 4.0 | 4.0 | 4.0 | 3.8 | 0.47 | 0.56 | 1.36 | 0.33 | 1.30 | 2.10 |
| 18 | 2.3 | 4.0 | 3.3 | 3.7 | 4.0 | 4.0 | 3.6 | -0.92 | -2.20 | -1.54 | 0.39 | -0.01 | 1.42 |
| 19 | 3.8 | 4.0 | 3.0 | 4.0 | 3.0 | 4.0 | 3.6 | 0.45 | 0.07 | 1.15 | -0.54 | -1.41 | 0.08 |
| 20 | 3.7 | 4.0 | 3.1 | 4.0 | 4.0 | 4.0 | 3.8 | 0.43 | 0.63 | 1.40 | 0.30 | 1.40 | 2.25 |
| 21 | 3.7 | 4.0 | 3.1 | 4.0 | 4.0 | 4.0 | 3.8 | 0.48 | 0.84 | 1.58 | 0.31 | 1.22 | 2.15 |
| 22 | 2.3 | 4.0 | 3.0 | 4.0 | 4.0 | 4.0 | 3.6 | -0.89 | -2.04 | -1.38 | 0.34 | 0.02 | 1.38 |
| 23 | 3.0 | 4.0 | 3.1 | 4.0 | 4.0 | 4.0 | 3.7 | -0.23 | -1.20 | -0.63 | 0.32 | 0.34 | 0.92 |
| 24 | 3.4 | 4.0 | 3.2 | 4.0 | 4.0 | 4.0 | 3.8 | 0.16 | -0.09 | 0.63 | 0.33 | 2.35 | 2.84 |
| 25 | 3.7 | 4.0 | 3.0 | 3.7 | 4.0 | 4.0 | 3.7 | 0.46 | 0.47 | 1.24 | 0.31 | 1.30 | 2.21 |
| 26 | 2.0 | 4.0 | 3.2 | 3.8 | 4.0 | 4.0 | 3.5 | -1.23 | -2.91 | -2.42 | 0.38 | -0.06 | 1.77 |
| 27 | 2.8 | 4.0 | 3.0 | 4.0 | 4.0 | 4.0 | 3.6 | -0.41 | -1.39 | -0.76 | 0.32 | 0.07 | 0.82 |
| 28 | 2.0 | 4.0 | 3.0 | 4.0 | 2.0 | 4.0 | 3.2 | -1.46 | -1.61 | 1.28 | -1.32 | -5.51 | -2.50 |
| 29 | 3.7 | 4.0 | 3.0 | 4.0 | 4.0 | 4.0 | 3.8 | 0.50 | 0.60 | 1.40 | 0.29 | 1.23 | 1.98 |
| 30 | 4.0 | 5.0 | 3.0 | 4.0 | 4.0 | 4.0 | 4.0 | 0.84 | 0.98 | 2.03 | -0.01 | 0.06 | 1.87 |
| 31 | 3.0 | 5.0 | 3.0 | 4.0 | 4.0 | 4.0 | 3.8 | -0.14 | -0.49 | 0.76 | 0.02 | -0.87 | 0.55 |
| 32 | 3.8 | 5.0 | 3.1 | 4.0 | 3.0 | 4.0 | 3.8 | 0.47 | 0.12 | 1.47 | -0.83 | -1.79 | -0.61 |
| 33 | 3.3 | 4.0 | 3.3 | 3.7 | 4.0 | 4.0 | 3.7 | 0.08 | -0.40 | 0.36 | 0.36 | 2.18 | 2.71 |
| 34 | 4.0 | 5.0 | 3.0 | 4.0 | 4.0 | 4.0 | 4.0 | 0.84 | 1.07 | 2.16 | -0.01 | -0.15 | 1.70 |
| 35 | 3.8 | 5.0 | 3.0 | 3.7 | 4.0 | 4.0 | 3.9 | 0.65 | 0.30 | 1.65 | 0.01 | -0.07 | 1.50 |
| 36 | 3.3 | 4.0 | 3.0 | 4.0 | 4.0 | 4.0 | 3.7 | 0.11 | -0.31 | 0.36 | 0.31 | 1.79 | 2.36 |
| 37 | 3.3 | 4.0 | 3.4 | 4.0 | 4.0 | 4.0 | 3.8 | 0.00 | -0.49 | 0.24 | 0.36 | 1.74 | 2.18 |
| 38 | 3.8 | 4.0 | 3.0 | 4.0 | 4.0 | 2.4 | 3.5 | 0.71 | -1.12 | 0.94 | -0.42 | -0.99 | 0.78 |

**Supplementary Table 3:** Deviations of **mean reproduction factor** of 38 mungbean genotypes tested at six environments from the corresponding environmental means. Genotype and environment scores corresponding to the PC1 and PC2 and at CLs @ 95 % enumerated through bootstrapping

| Sl No. | **SMT** | **KN** | **BUB** | **AH** | **DP** | **JH** | **Mean** | **PC1** | **Lower limit** | **Upper limit** | **PC2** | **Lower limit** | **Upper limit** |
| --- | --- | --- | --- | --- | --- | --- | --- | --- | --- | --- | --- | --- | --- |
| 1 | 1.91 | 2.14 | 0.61 | 1.76 | 0.98 | 1.98 | 1.56 | 0.15 | 0.38 | 1.95 | 0.32 | -0.50 | 0.87 |
| 2 | 0.92 | 1.68 | 0.69 | 1.64 | 1.85 | 1.77 | 1.42 | -0.62 | -2.20 | -0.85 | -0.32 | -1.22 | -0.57 |
| 3 | 0.85 | 1.69 | 0.65 | 1.57 | 1.78 | 2.09 | 1.44 | -0.65 | -2.21 | -0.81 | -0.53 | -2.03 | -1.42 |
| 4 | 1.58 | 1.68 | 0.75 | 1.80 | 2.12 | 1.91 | 1.64 | -0.52 | -2.18 | -1.14 | 0.15 | 0.62 | 1.25 |
| 5 | 1.89 | 1.88 | 0.38 | 1.76 | 0.91 | 2.11 | 1.49 | -0.09 | -0.07 | 1.82 | 0.33 | -0.93 | 0.71 |
| 6 | 1.84 | 2.73 | 0.83 | 1.91 | 1.65 | 2.14 | 1.85 | 0.57 | 1.27 | 2.31 | -0.01 | -0.24 | 0.73 |
| 7 | 0.41 | 3.89 | 0.58 | 1.82 | 0.95 | 0.51 | 1.36 | 1.60 | -0.86 | 2.25 | -0.71 | -2.76 | -0.24 |
| 8 | 0.74 | 1.69 | 0.92 | 1.85 | 1.87 | 2.51 | 1.60 | -0.77 | -2.29 | -0.55 | -0.82 | -2.42 | -1.65 |
| 9 | 1.26 | 2.21 | 0.33 | 1.77 | 2.37 | 1.49 | 1.57 | -0.07 | -1.25 | 0.08 | -0.05 | -0.52 | 0.55 |
| 10 | 2.01 | 1.37 | 0.66 | 1.90 | 1.87 | 1.34 | 1.52 | -0.61 | -1.95 | -0.33 | 0.89 | 2.67 | 3.18 |
| 11 | 2.75 | 2.25 | 0.84 | 2.11 | 0.91 | 0.52 | 1.56 | 0.62 | -0.04 | 2.13 | 1.70 | 1.51 | 3.04 |
| 12 | 1.07 | 3.98 | 0.87 | 1.54 | 1.01 | 1.67 | 1.69 | 1.72 | 2.35 | 4.96 | -0.78 | -3.36 | -1.56 |
| 13 | 1.82 | 2.95 | 0.28 | 1.70 | 1.93 | 0.64 | 1.55 | 0.94 | 0.39 | 1.91 | 0.62 | -0.24 | 1.24 |
| 14 | 0.77 | 1.65 | 0.64 | 1.79 | 1.89 | 2.21 | 1.49 | -0.76 | -2.45 | -0.92 | -0.63 | -2.05 | -1.37 |
| 15 | 1.55 | 1.37 | 0.77 | 2.10 | 1.68 | 1.82 | 1.55 | -0.77 | -2.73 | -1.44 | 0.30 | 0.77 | 1.61 |
| 16 | 0.73 | 1.23 | 0.82 | 2.28 | 1.96 | 2.17 | 1.53 | -1.21 | -3.74 | -1.95 | -0.51 | -1.67 | -0.65 |
| 17 | 1.86 | 1.51 | 0.85 | 1.98 | 2.07 | 1.74 | 1.67 | -0.61 | -2.02 | -0.66 | 0.53 | 1.89 | 2.46 |
| 18 | 0.64 | 2.79 | 0.93 | 1.46 | 2.46 | 2.67 | 1.82 | 0.17 | -0.54 | 1.31 | -1.35 | -2.56 | -1.12 |
| 19 | 2.06 | 1.72 | 0.80 | 2.00 | 0.88 | 1.81 | 1.54 | -0.19 | -0.34 | 1.36 | 0.65 | -0.22 | 1.16 |
| 20 | 1.78 | 1.78 | 0.80 | 1.56 | 2.06 | 2.20 | 1.70 | -0.39 | -1.12 | -0.11 | 0.14 | 0.73 | 1.50 |
| 21 | 1.91 | 1.58 | 0.77 | 2.07 | 2.44 | 1.79 | 1.76 | -0.58 | -1.87 | -0.51 | 0.52 | 1.02 | 1.87 |
| 22 | 0.69 | 3.03 | 0.60 | 2.28 | 2.16 | 2.35 | 1.85 | 0.44 | 0.02 | 1.68 | -1.14 | -2.93 | -1.82 |
| 23 | 1.24 | 1.37 | 0.56 | 2.26 | 1.58 | 2.11 | 1.52 | -0.87 | -2.86 | -1.62 | -0.07 | -0.60 | 0.31 |
| 24 | 1.61 | 1.58 | 0.75 | 2.21 | 2.39 | 1.99 | 1.76 | -0.68 | -2.66 | -1.21 | 0.19 | 0.47 | 1.39 |
| 25 | 1.84 | 2.21 | 0.65 | 1.85 | 1.96 | 1.88 | 1.73 | 0.07 | 0.04 | 0.88 | 0.25 | 0.86 | 1.42 |
| 26 | 0.43 | 1.63 | 0.85 | 1.60 | 1.60 | 1.70 | 1.30 | -0.75 | -1.64 | 0.23 | -0.68 | -1.91 | -0.94 |
| 27 | 0.96 | 1.76 | 0.68 | 1.74 | 1.95 | 2.84 | 1.66 | -0.68 | -1.39 | 0.10 | -0.82 | -1.96 | -0.89 |
| 28 | 0.36 | 1.35 | 0.84 | 1.86 | 0.35 | 1.49 | 1.04 | -0.86 | -0.80 | 1.15 | -0.48 | -2.22 | 0.00 |
| 29 | 1.89 | 1.79 | 0.85 | 1.76 | 1.60 | 1.70 | 1.60 | -0.25 | -0.76 | 0.10 | 0.50 | 1.49 | 2.01 |
| 30 | 2.85 | 3.83 | 0.59 | 1.64 | 1.82 | 1.88 | 2.10 | 1.90 | 2.20 | 4.96 | 0.62 | 0.74 | 2.68 |
| 31 | 1.23 | 3.75 | 0.84 | 1.97 | 2.47 | 1.81 | 2.01 | 1.30 | 1.19 | 3.40 | -0.67 | -1.84 | 0.10 |
| 32 | 1.84 | 3.72 | 0.49 | 1.60 | 1.07 | 2.05 | 1.80 | 1.63 | 4.63 | 6.41 | -0.23 | -1.50 | 0.17 |
| 33 | 1.57 | 1.72 | 0.97 | 1.60 | 2.03 | 2.47 | 1.73 | -0.53 | -1.69 | -0.59 | -0.15 | 0.05 | 1.03 |
| 34 | 2.87 | 4.22 | 0.70 | 1.66 | 1.71 | 2.03 | 2.20 | 2.26 | 3.57 | 6.75 | 0.46 | 0.50 | 2.66 |
| 35 | 1.83 | 1.86 | 0.56 | 1.62 | 1.58 | 1.46 | 1.49 | -0.13 | -0.70 | 0.20 | 0.54 | 1.36 | 2.07 |
| 36 | 1.52 | 1.51 | 0.86 | 1.99 | 2.22 | 1.58 | 1.61 | -0.69 | -2.38 | -0.94 | 0.31 | 0.68 | 1.43 |
| 37 | 1.46 | 2.97 | 1.07 | 2.02 | 2.10 | 1.88 | 1.92 | 0.64 | 1.23 | 2.37 | -0.29 | -0.73 | 0.31 |
| 38 | 1.87 | 1.29 | 0.83 | 2.04 | 2.83 | 0.39 | 1.54 | -0.76 | -2.54 | 0.96 | 1.22 | -0.63 | 1.51 |

**Supplementary Table 4: Direct (Diagonal) and indirect effect of environment variables on nematode score**

| **Characters** | **Elevation** | **Avg. Rainfall** | **RH** | **Min Temp** | **Max Temp** |
| --- | --- | --- | --- | --- | --- |
| **Elevation** | **1.284** | -0.575 | 2.895 | -2.224 | 1.598 |
| **Avg. Rainfall** | 0.295 | **2.501** | -2.277 | -0.417 | -0.202 |
| **RH** | 0.963 | 1.475 | **-3.860** | 1.918 | -1.077 |
| **Min Temp** | 1.027 | -0.375 | -2.66 | **2.779** | -1.278 |
| **Max Temp** | -1.220 | -0.300 | 2.470 | -2.112 | **1.682** |

**Residual Effect= 0.08070623**
